# White matter integrity and structural brain network topology in cerebral small vessel disease: The Hamburg city health study

**DOI:** 10.1002/hbm.25301

**Published:** 2020-12-08

**Authors:** Benedikt M. Frey, Marvin Petersen, Eckhard Schlemm, Carola Mayer, Uta Hanning, Kristin Engelke, Jens Fiehler, Katrin Borof, Annika Jagodzinski, Christian Gerloff, Götz Thomalla, Bastian Cheng

**Affiliations:** ^1^ Department of Neurology University Medical Center Hamburg‐Eppendorf Hamburg Germany; ^2^ Department of Diagnostic and Interventional Neuroradiology University Medical Center Hamburg‐Eppendorf Hamburg Germany; ^3^ Epidemiological study center University Medical Center Hamburg‐Eppendorf Hamburg Germany; ^4^ Department of General and Interventional Cardiology University Heart and Vascular Center Hamburg Germany

**Keywords:** cerebral small vessel disease, structural brain networks, peak width of skeletonized mean diffusivity, white matter hyperintensities of presumed vascular origin, diffusion weighted imaging, topological brain network disturbances

## Abstract

Cerebral small vessel disease is a common finding in the elderly and associated with various clinical sequelae. Previous studies suggest disturbances in the integration capabilities of structural brain networks as a mediating link between imaging and clinical presentations. To what extent cerebral small vessel disease might interfere with other measures of global network topology is not well understood. Connectomes were reconstructed via diffusion weighted imaging in a sample of 930 participants from a population based epidemiologic study. Linear models were fitted testing for an association of graph‐theoretical measures reflecting integration and segregation with both the Peak width of Skeletonized Mean Diffusivity (PSMD) and the load of white matter hyperintensities of presumed vascular origin (WMH). The latter were subdivided in periventricular and deep for an analysis of localisation‐dependent correlations of cerebral small vessel disease. The median WMH volume was 0.6 mL (1.4) and the median PSMD 2.18 mm^2^/s x 10^−4^ (0.5). The connectomes showed a median density of 0.880 (0.030), the median values for normalised global efficiency, normalised clustering coefficient, modularity Q and small‐world propensity were 0.780 (0.045), 1.182 (0.034), 0.593 (0.026) and 0.876 (0.040) respectively. An increasing burden of cerebral small vessel disease was significantly associated with a decreased integration and increased segregation and thus decreased small‐worldness of structural brain networks. Even in rather healthy subjects increased cerebral small vessel disease burden is accompanied by topological brain network disturbances. Segregation parameters and small‐worldness might as well contribute to the understanding of the known clinical sequelae of cerebral small vessel disease.

## Graphical abstract

Even in rather healthy subjects increased cerebral small vessel disease burden is accompanied by topological brain network disturbances. Besides the known mediation effect of integration parameters, the segregation parameters might as well contribute to the understanding of the known clinical sequelae of cerebral small vessel disease.

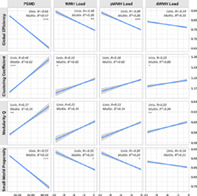

## 1 INTRODUCTION

White matter hyperintensities of presumed vascular origin (WMH) are a common finding in MRI of elderly people and are a hallmark of cerebral small vessel disease (CSVD) (Wardlaw et al., 2013). CSVD is considered to result from damage to small perforating arteries, arterioles, capillaries and venules of the human brain and represents an increasing burden in the ageing societies of industrialised countries (Pantoni, 2010; ter Telgte et al., 2018). Besides rare hereditary causes, the major underlying pathology is arteriolosclerosis due to age and cardiovascular risk factors (Pantoni, 2010). The clinical importance of CSVD lies in its association with various clinical sequelae such as ischaemic and haemorrhagic stroke, cognitive decline, dementia, late‐life depression as well as gait and urinary complaints (Frey et al., 2019; Pantoni, 2010; Rensma, van Sloten, Launer, & Stehouwer, 2018; ter Telgte et al., 2018).

Findings from structural magnetic resonance imaging play a pivotal role in defining CSVD. The most common surrogate marker is the extent of WMH. However, this measure has some weaknesses such as the error‐prone quantitative assessment of WMH via automated segmentation (Frey et al., 2019) as well as rather weak correlation with clinical symptoms (Baykara et al., 2016). A novel surrogate marker of CSVD called “peak width of skeletonised mean diffusivity” (PSMD) was suggested to overcome aforementioned limitations due to the robust nature of its computation and evidently strong correlations with clinical symptoms (Baykara et al., 2016; Wei et al., 2019). The PSMD measures the distribution width of the mean diffusivity, that is, the mean diffusion of water in all directions calculated by the diffusion tensor, in the white matter and thereby assesses the microstructural properties of the tissue. As WMH represent the part of the pathologic process visible on conventional MRI and thereby presumably only the tissue worst affected by CSVD, the PSMD particularly might be more sensitive to rather subtle pathologic changes in the microstructure of the brains white matter.

In recent years, growing evidence supports the understanding of the brain as a complex network of interconnected areas. The structural connectome as a comprehensive map of neuronal connections interprets the brain as a network based on two components: Nodes, which represent prespecified cortical areas; and edges, representing the interconnecting white matter tracts (Fornito & Bullmore, 2015). Connectomes allow inferences about the structural organisation and integrity of the human brain via graph theoretical analysis with global graph parameters reflecting topological network characteristics. As such, measures of segregation (e.g., the clustering coefficient and the modularity Q) reflect on the network's capability of distributed and parallel information processing, whereas measures of integration (e.g., global and local efficiency) give hints about the brain's capacities of combining information from such distributed processes.

The analysis of these global topological characteristics allows for the investigation of pathologic alterations in the structural network of the brain underlying neurological conditions like Alzheimer's, stroke and multiple sclerosis (Crofts et al., 2011; Stam, 2014; Stellmann et al., 2017). In the context of CSVD, previous studies suggest disturbed topological network properties as a possible mechanism underlying clinical presentations in these patients (ter Telgte et al., 2018). Decreased integration parameters were observed in particular and related to for example, cognitive performance of the participants (Lawrence, Chung, Morris, Markus, & Barrick, 2014; Reijmer et al., 2015; Tuladhar et al., 2016b). However, to what extent CSVD affects the degree of segregation in structural network topologies is less comprehensively studied.

The aim of this study was to investigate how brain network topology is affected by CSVD, namely the association of topological network parameters with the PSMD as a novel and robust marker of white matter integrity in CSVD and WMH load as the most common imaging markers representing CSVD. In addition, a deeper understanding of localisation‐dependent correlations of CSVD was pursued by subdivision of WMH in periventricular (pWMH) and deep (dWMH).

## 2 METHODS

### 2.1 Study design and participants

The Hamburg City Health Study (HCHS) is a single centre, prospective, epidemiologic cohort study with emphasis on imaging to improve the identification of individuals at risk for major chronic diseases and to improve early diagnosis and survival. A detailed description of the overall study design was published separately (Jagodzinski et al., 2019). In summary, of all inhabitants living in Hamburg, a random sample is drawn from a total of 45,000 (aged 45–74) based on the official inhabitant data files. A written invitation to participate in HCHS is sent to their home address. All individuals willing to participate are invited to a baseline visit where they undergo an extensive assessment of their cardiovascular history and status. Of these, all participants with a Framingham stroke risk score (FSRS) of >7 points are invited to additional brain scans (Aparicio et al., 2017). Furthermore, 1,500 healthy participants are selected for a control group. For an explorative analysis of cognitive functions, results from the „Mini Mental Status Test “(MMST), the Trail Making Test part A (TMTA) and the Trail Making Test part B (TMTB) were selected. In addition, age, sex and years of educations were selected for analysis. For the present study, the MRI datasets of the first 1,000 participants undergoing imaging studies were selected ‐ independent of FSRS. The local ethics committee approved the HCHS, and written informed consent was obtained from all participants.

### 2.2 MRI acquisition

Images were acquired using a 3‐T Siemens Skyra MRI scanner (Siemens, Erlangen, Germany). For single‐shell diffusion weighted imaging (DWI), 75 axial slices were obtained covering the whole brain with gradients (b = 1,000 s/mm2) applied along 64 noncollinear directions with the following sequence parameters: repetition time (TR) = 8,500 ms, echo time (TE) = 75 ms, slice thickness (ST) = 2 mm, in‐plane resolution (IPR) = 2 × 2 mm, anterior–posterior phase‐encoding direction. For 3D T1‐weighted anatomical images, rapid acquisition gradient‐echo sequence (MPRAGE) was used with the following sequence parameters: TR = 2,500 ms, TE = 2.12 ms, 256 axial slices, ST = 0.94 mm, and IPR = 0.83 × 0.83 mm. 3D T2‐weighted fluid attenuated inversion recovery (FLAIR) images were measured with the following sequence parameters: TR = 4,700 ms, TE = 392 ms, 192 axial slices, ST = 0.9 mm, and IPR = 0.75 × 0.75 mm.

### 2.3 Quantification of CSVD

For segmentation of WMH, we used FSLs Brain Intensity AbNormality Classification Algorithm (BIANCA) (Griffanti et al., 2016), a fully automated, supervised k‐nearest neighbour (k‐NN) algorithm. The training dataset comprised masks of WMH for the first 100 participants. These masks were derived by selecting only the voxels that had been identified as WMH by two trained raters (MP and CM) independently with manual segmentation. The mean Dice Similarity Index between the segmentation of both raters was 0.63.

Derived masks of WMH were divided into periventricular (pWMH) and deep (dWMH) by a 10 mm distance threshold to the ventricles (DeCarli, Fletcher, Ramey, Harvey, & Jagust, 2005; Griffanti et al., 2018). WMH load was calculated as the share of WMH in the brain tissue volume (intracranial volume ‐ ventricle volume) and logarithmised for further statistical analysis due to a right‐skewed distribution. Logarithmic pWMH respectively dWMH load were calculated analogously.

PSMD was calculated with the available original scripts on diffusion tensor imaging (DTI) data (http://www.psmd-marker.com, (Baykara et al., 2016)). In brief, the precalculated maps of MD were brought to MNI space using the coregistration of the precalculated FA maps with the FSL‐TBSS package (Smith et al., 2006). Following white matter tract skeletonisation of the standardised MD maps, the PSMD was calculated via histogram analysis.

### 2.4 Connectome reconstruction

All imaging data was processed using MRtrix 3.0 ((Tournier et al., 2019), http://www.mrtrix.org), Advanced Normalisation Tools (ANTs, https://github.com/ANTsX/ANTs), the FMRIB Software Library 5.0.10 (FSL, https://fsl.fmrib.ox.ac.uk) and FreeSurfer 6.0 (https://surfer.nmr.mgh.harvard.edu). Network nodes were defined by parcellation of the grey matter areas in T1w according to the Desikan‐Killiany and Aseg atlas (Desikan et al., 2006; Filipek, Richelme, Kennedy, & Caviness, 1994) including a total of 84 cortical and subcortical regions. There was no anatomical overlap between brain regions considered as nodes in the analysis. DWI preprocessing involved denoising, removal of Gibbs ringing artefacts, eddy current correction and motion correction, bias field correction as well as susceptibility distortion correction based on nonlinear registration (Andersson & Sotiropoulos, 2016; Avants, Epstein, Grossman, & Gee, 2008; Kellner, Dhital, Kiselev, & Reisert, 2016; Tustison et al., 2010; Veraart et al., 2016). Constrained spherical deconvolution and anatomically constrained tractography (ACT) (Smith, Tournier, Calamante, & Connelly, 2012) allowed for streamlines reconstruction from preprocessed diffusion images. Upon that streamlines were filtered by spherical deconvolution informed filtering of tractograms (SIFT2) (Smith, Tournier, Calamante, & Connelly, 2015). Two nodes were assumed to be connected by an edge if DWI signal‐derived streamlines were running between them. The edge weight was determined by the weighted streamline count reaching from one node to the other. The detailed pipeline and an illustration of regions of interests derived from the atlases and used for node definition can be found in the supplementary materials.

### 2.5 Connectome analysis

Connectomes produced were further processed with the brain connectivity (BCT) toolbox (Rubinov & Sporns, 2010) in matlab (v2018b). This included connectome normalisation and global graph parameter computation. The following topological global graph parameters have been extracted: global efficiency, clustering coefficient, modularity Q and small‐world propensity. The graph parameters are explained in Box 1. The global efficiency and clustering coefficient are sensitive to low level features of the connectomes as they are derived from the value of connection weights and degree distribution. To account for this dependency, both were normalised against graph parameters derived from and averaged over 100 null models which were acquired by randomly rewiring subject connectomes preserving the degree distribution (Maslov, Sneppen, & Zaliznyak, 2002). Corresponding standard errors are listed in the results. Furthermore, the network density, that is, the fraction of present and possible connections, and median edge weight, that is, the median of all connection weights, were computed for all participants.

**Global graph parameters derived in this study**

Weighted clustering coefficient (Onnela, Saramäki, Kertész, & Kaski, 2005; Watts & Strogatz, 1998).

The weighted clustering coefficient of a node is defined as the normalised sum of geometrically averaged edge weights of all triangles associated with the node. Thus, the average of this parameter over all nodes indicates how intensively the network is locally interconnected and thereby reflects its capability of segregated computation.

Modularity Q (Newman, 2006).

The modularity Q is the result of an iterative optimization process. First, partition of the connectome in nonoverlapping modules ‐ i.e., highly interconnected subgroups of nodes ‐ is performed by applying Newman's spectral community detection. Upon that the modularity Q is calculated: for each module the weighted count of edges present within the module is surveyed and subtracted by the weighted edge count expected by chance. Subsequently, all module specific values are summed up resulting in Q. Hence, a positive Q indicates a higher intramodular connectivity than expected by chance, indicating modular structure. Based on the former partition a new partition is defined by an optimization algorithm and this process is reiterated until Q does not increase anymore. This maximal Q is the value reported.

Weighted global efficiency (Latora & Marchiori, 2001).

The global efficiency is defined as the average inverse shortest path length. The length of a path between two nodes is the total weight of edges comprising that path.

Small‐world propensity {Muldoon et al., 2016}.

The small‐world propensity allows for quantification of small‐world structure in weighted and dense networks: it indicates how much a networks clustering coefficient and shortest path length deviate from random and lattice null models with the same node amount and degree distribution and relates both deviations. Thus, the parameter provides insights about the degree a network exhibits parallel presence of strong integration and segregation characteristics. A network with a small‐world propensity above 0.6 is considered to show pronounced small‐world structure.

### 2.6 Statistical analysis

The statistical analysis has been performed in R (v3.1.4). To assess the associations of the CSVD surrogate markers with global graph parameters, a linear regression analysis was performed. For facilitated interpretability effects of simple linear regressions are conveyed in the first place. Subsequently, we report whether significance remains after correcting for age, sex, brain volume and median edge weight. To compare the correlations of pWMH and dWMH regarding the global graph parameters, Pearson and Filon's z was applied (Diedenhofen & Musch, 2015). For an explorative analysis of associations with cognitive functions, simple and multivariable linear regression models adjusted for age, sex and years of education were fitted for the association between performance in MMST, TMT‐A and TMT‐B with global graph parameters and CSVD surrogate markers, respectively. Unless stated otherwise, descriptive statistics are given as median with interquartile range (IQR).

### 2.7 Data assessment and quality assurance

The quality of the initial data and the reliability of the relevant processing steps in our pipeline were assessed repeatedly. DWI‐data was initially checked for completeness of the sequence and signal‐to‐noise ratio, mean voxel intensity, outlier count and maximum voxel intensity outlier count were calculated (Roalf et al., 2016). Finally, the output statistics of the outlier replacement step of FSL's eddy were used for additionally identifying data with poor quality.

## 3 RESULTS

### 3.1 Sample characteristics

**FIGURE 1:**
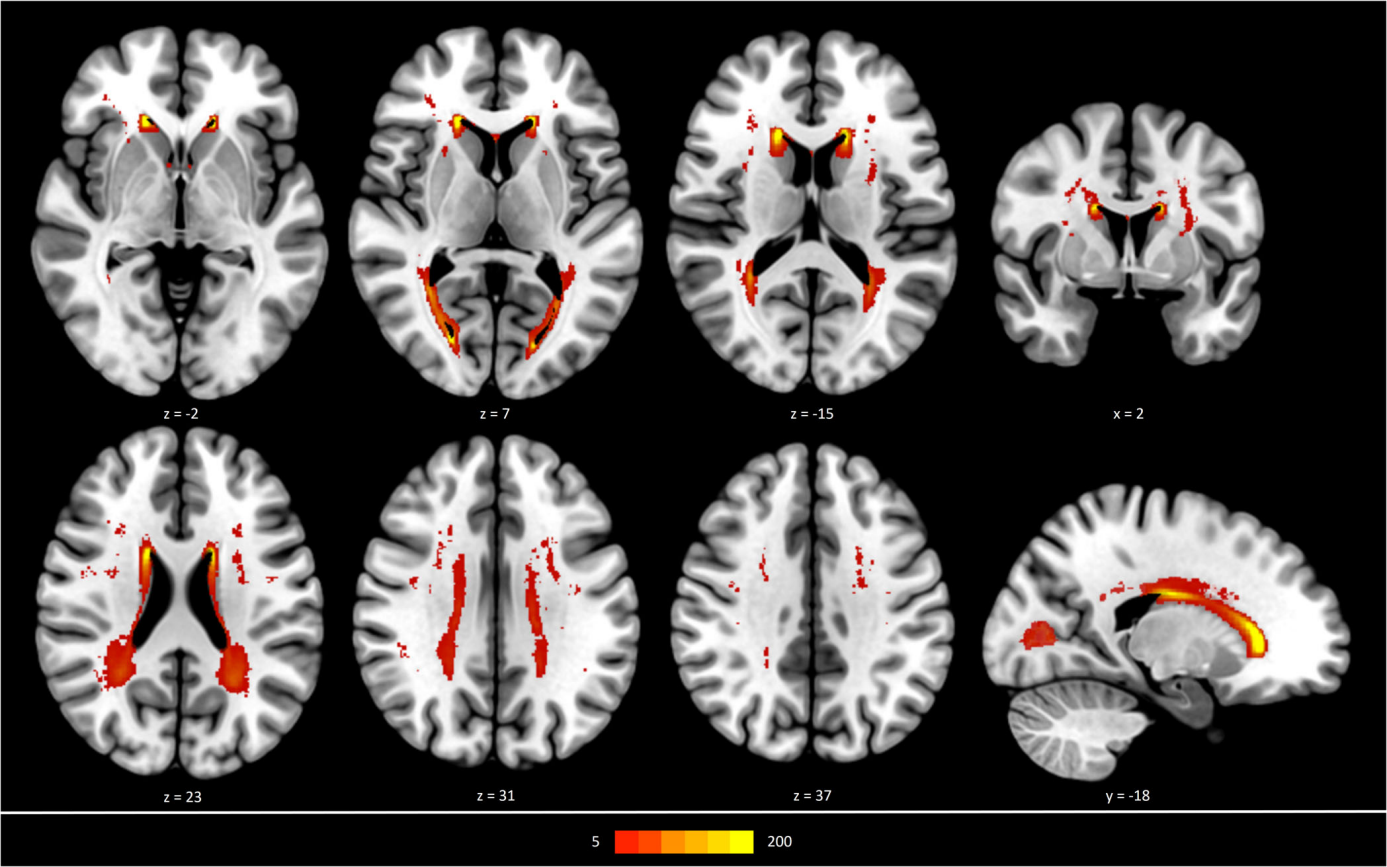
*Distribution of White Matter Hyperintensities (WMH) in a cohort of 930 participants*. The map presents the frequency of WMH in a specific voxel as indicated by the coloured bar and superimposed on a standard brain template in MNI‐152 space

Descriptive statistics are listed in Table 1. Due to missing data and after quality assessment, the final sample for this study comprised 930 participants. 21 participants were excluded due to missing imaging data, 40 participants were excluded due to poor quality or incompleteness of imaging data (39 DWI, 1 FLAIR) and 9 participants were excluded due to poor quality of certain processing steps. The median subject age was 64 years (IQR = 14), 45.6% were women. Figure 1 depicts a heatmap delineating the spatial distribution of WMH, the median WMH volume was 0.6 mL (1.4) and the median PSMD 2.18 mm2/s x 10^−4^ (0.5). The structural connectomes showed a median density of 0.880 (0.030), the median values for normalised global efficiency, normalised clustering coefficient, modularity Q and small‐world propensity were 0.780 (0.045), 1.182 (0.034), 0.593 (0.026) and 0.876 (0.040) respectively. The median score for the MMST was 28 points (2), the median time for the TMTA was 36 seconds (17) and 79 seconds (37) for the TMTB. A mean connectome matrix can be found in the supplementary materials.

**TABLE 1:** characteristics of the study sample

Variable	Count
Sex = female	424 (45.6%)
Age (years), median (IQR)	64 (14)
Vascular risk factors
History of smoking, n (%)	560 (66.1%)
History of hypertension, n (%)	637 (72.0%)
Diabetes, n (%)	74 (8.7%)
Body‐mass‐index, median (IQR)	26.2 (5.5)
Conventional MRI measures	
Brain volume (ml), median (IQR)	1,483.7 (203.1)
Ventricle volume (ml), median (IQR)	25.4 (18.4)
WMH volume (ml), median (IQR)	0.6 (1.4)
pWMH volume (ml), median (IQR)	0.5 (1.1)
dWMH volume (ml), median (IQR)	0.1 (0.2)
WMH load (%), median (IQR)	0.04 (0.09)
pWMH load (%), median (IQR)	0.03 (0.08)
dWMH load (%), median (IQR)	0.01 (0.01)
PSMD (mm2/s x 10^−4^), median (IQR)	2.18 (0.5)
Graph theoretical network measures	
Edge strength, median (IQR)	28.1 (9.5)
Network density, median (IQR)	0.880 (0.030)
Norm. Global efficiency, median (IQR)	0.780 (0.045)
Standard error for null model global efficiency, median (IQR)	0.001 (<0.001)
Norm. Clustering coefficient, median (IQR)	1.182 (0.034)
Standard error for null model clustering coefficient, median (IQR)	<0.001 (<0.001)
Modularity Q, median (IQR)	0.593 (0.026)
Small‐world propensity, median (IQR)	0.876 (0.040)

*Note*: Data was partly unavailable for the following variables (n): History of smoking (83), History of hypertension (45), Diabetes (80), Body‐Mass‐Index (56).

Abbreviations: PSMD, peak width of skeletonised mean diffusivity; WMH, total white matter hyperintensities; pWMH, periventricular white matter hyperintensities; dWMH, deep white matter hyperintensities; Norm., normalized.

### 3.2 Association of CSVD surrogate markers with global graph parameters

**FIGURE 2:**
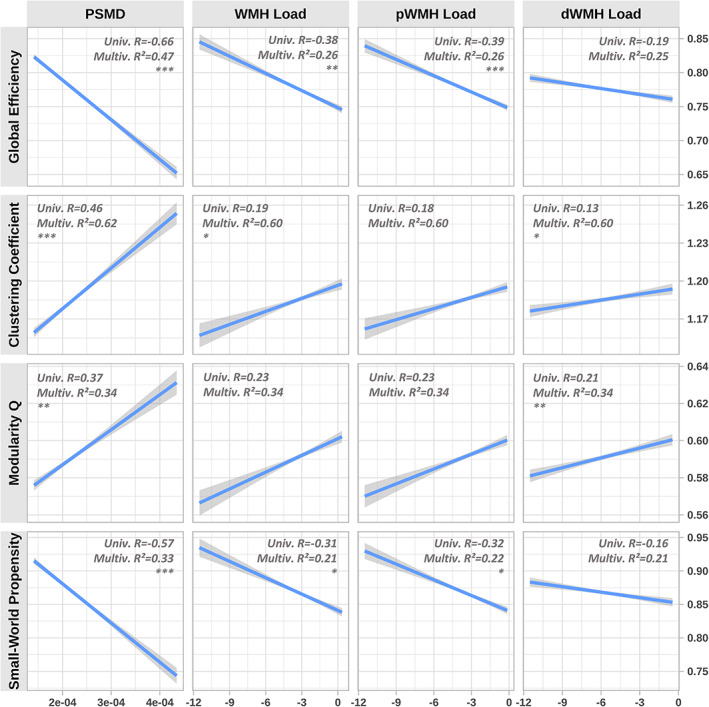
Association of CSVD surrogate markers with global graph parameters. Results of simple linear regression modelling are illustrated using CSVD surrogate markers as independent variables (columns) and global topological graph parameters as dependent variables (rows). Grey bands represent confidence intervals. Correlation (R) from simple correlation analysis and explained variance (R^2^) from multivariable models are reported. Asterisks indicate level of significance after inclusion of covariates (* = *p* < .05, ** = *p* < .01, *** = *p* < .001). PSMD = peak width of skeletonised mean diffusivity, WMH = total White Matter Hyperintensities, pWMH = periventricular White Matter Hyperintensities, dWMH = deep White Matter Hyperintensities

Simple linear regression analysis of the relationship between CSVD surrogate markers and global graph parameters revealed consistent and statistically significant correlations which are illustrated in Figure 2. The normalised global efficiency (R = −0.66, *p* < .001) and small‐world propensity (R = −0.57, *p* < .001) were negatively correlated with PSMD. Normalised clustering coefficient (R = 0.46, *p* < .001) and modularity Q (R = 0.37, *p* < .001) were positively correlated with PSMD. Correlations of log WMH load, log pWMH load and log dWMH load with global graph parameters exhibited smaller effect sizes and significance levels. After correcting for age, sex, brain tissue volume, median edge weight and cardiovascular risk factors, all but the following correlations remained significant: WMH load with respect to the modularity (*p* = .115), dWMH load with respect to global efficiency (*p* = .422) and small‐world propensity (*p* = .614) as well as pWMH load with respect to clustering coefficient (*p* = .104) and modularity (*p* = .155) remained significant. Applying Pearson and Filon's Z for correlation comparison revealed that global efficiency (R = −0.39 vs R = −0.19, *p* < .001) as well as small world propensity (R = −0.32 vs R = −0.16, *p* < .001) correlated significantly stronger with pWMH load than with dWMH load. Correlations of pWMH and dWMH with the normalised clustering coefficient (*p* = .177) and modularity Q (*p* = .398) showed no statistically significant difference. Further details of the linear regression results are listed in the supplementary materials.

### 3.3 Analysis of cognitive function, CSVD surrogate markers and global graph parameters

MMST was found to be associated with PSMD (simple regression analysis *p* = .017 and multivariable analysis *p* = .244, respectively), pWMH (*p* = .020 and *p* = .789, respectively), global efficiency (*p* = .019 and *p* = .599, respectively) and modularity Q (*p* = .013 and *p* = .281, respectively). There was no association of MMST and dWMH (*p* = .191 and *p* = .956, respectively), Clustering coefficient (*p* = .263 and *p* = .957, respectively) and small‐world propensity (*p* = .053 and *p* = .637, respectively).

TMTA was found to be associated with PSMD (*p* < .001 and *p* = .021, respectively), pWMH (*p* < .001 and *p* = .059, respectively), dWMH (*p* < .001 and *p* = .005, respectively), global efficiency (*p* < .001 and *p* = .009, respectively), modularity Q (*p* < .001 and *p* = .059, respectively) and small‐world propensity (*p* < .001 and *p* = .002, respectively). There was no association of TMTA and Clustering coefficient (*p* = .083 and *p* = .877, respectively). After correcting for multiple testing, only the association with small‐world propensity remained significant.

TMTB was found to be associated with PSMD (*p* < .001 and *p* < .001, respectively), pWMH (*p* < .001 and *p* = .315, respectively), dWMH (*p* < .001 and *p* = .185, respectively), global efficiency (*p* < .001 and *p* = .006, respectively), Clustering coefficient (*p* < .001 and *p* = .058, respectively), modularity Q (*p* < .001 and *p* = .014, respectively) and small‐world propensity (*p* < .001 and *p* < .001, respectively). After correcting for multiple testing, only the association with small‐world propensity and PSMD remained significant.

## 4 DISCUSSION

In this analysis of a population‐based sample of 930 subjects, we investigated the association of CSVD with brain network topology using state of the art methodology to reconstruct structural connectomes derived from DWI and structural imaging data. As a main finding, we identified a significant association of CSVD burden with decreased integration and increased segregation of structural brain networks resulting in a weakened small‐world structure. These findings provide novel insights into the effects of CSVD on the architecture and integrity of brain networks and may foster the understanding of the clinical sequelae of CSVD.

### 4.1 Association of CSVD with global graph parameters

Recent research provided evidence about the topological properties of the underlying network organisation of the human brain (Latora & Marchiori, 2001; Sporns & Zwi, 2004). Accordingly, the human brain is organised in a topological paradigm called “small world” as it applies to most real‐world networks (Costa et al., 2011). Small‐worldness describes a compromise between pronounced segregation, meaning that distributed and specialised processing happens in subsections of the brain, and integration, the brain's capacity of integrating information from distributed processes (Watts & Strogatz, 1998). Hence a networks small‐worldness is immediately depending on sufficient integration and segregation characteristics. To investigate the network topology of the reconstructed connectomes and its alteration by CSVD, topological network parameters were assessed and related to PSMD as a novel and robust surrogate parameter of white matter integrity and WMH load as the most common imaging marker representing CSVD. The investigated connectomes were of strong small‐worldness as the median small‐world propensity of 0.87 was above the suggested threshold of 0.6 (Muldoon, Bridgeford, & Bassett, 2016). It turned out that the integration parameter ‐ global efficiency ‐ decreases while the segregation parameters ‐ clustering coefficient and modularity Q ‐ increase with higher CSVD burden. Both phenomena ultimately result in weakening of small‐world topology as a relatively low small‐world propensity accompanies high CSVD burden. To put this into context, the observed alterations of brain network topology might suggest that the brain's capacity of integrating distributed information decreases, while the capability of distributed computation increases with higher CSVD burden. We found that periventricular WMH (pWMH) drive the reduction of integration capabilities and small‐worldness overall, showing significantly higher effects than deep white matter WMH (dWMH). The effects of dWMH on segregation parameters were of statistical significance whereas the effects of pWMH were not. Since long‐range connections are preferentially passing through ventricle‐near regions (Brodal, 2016), the observed topological alterations might be explained by a disproportionate decrease of long‐range connections in CSVD as suggested in previous studies in this cohort of participants of the HCHS (Petersen et al., 2020).

Global efficiency depends on the integrity of these long‐distance connections, as these connections enable communication of remote brain regions (Watts & Strogatz, 1998). Hence a reduction of these fibres might explain the reduced global efficiency we observed in subjects with high CSVD burden. This is in line with the hypothesis that primarily pWMH are responsible for global efficiency decline and therefore the cognitive decline in CSVD patients, as supported by other studies (Cees De Groot et al., 2000).

The increased clustering coefficient and modularity might be attributable to affected long‐range connections as well. Provided they are underrepresented, long‐range connections might lead with higher probability to open triangles, respectively less modular structure, decreasing the average clustering coefficient and modularity. Conversely, less long‐range connections might yield higher segregation parameters. Moreover, in presence of CSVD pathology the normal appearing white matter (NAWM) fibre density might increase compensatorily yielding more intensively connected node neighbourhoods and modules, as hypothesised in stroke patients (Crofts et al., 2011; Dancause et al., 2005).

### 4.2 Importance of global graph parameters for clinical sequelae of CSVD

These findings are of clinical relevance hence they might illuminate how associations between CSVD and its known clinical sequelae are mediated. A lower global efficiency, regardless of health status, is associated with poorer cognitive performance (Bassett et al., 2009; Berlot, Metzler‐Baddeley, Ikram, Jones, & O'Sullivan, 2016; Heuvel, Stam, Kahn, & Pol, 2009; Li et al., 2009). In line with this, recent studies found that the interrelation of CSVD occurrence and cognitive decline is mediated by a decreased global efficiency (Lawrence et al., 2014; Tuladhar et al., 2016a; Tuladhar, van Uden, et al., 2016b). In our analysis, we detected an association between a lower extent of small‐world characteristics in structural brain networks and worse performance in the Trail Making Test. This observation is in line with previously reported alterations of large‐scale brain networks and cognitive deficits. However, our results have to be considered as exploratory. Additional statistical models including several additional mediating factors are needed in future studies.

Looking beyond cognition, results from previous cohort studies back the assumption that late‐life depression is associated with CSVD, summarised as the vascular depression hypothesis (Alexopoulos, 2006). Depressive CSVD patients were found to have a significantly decreased global efficiency compared to patients without depressive symptoms (Xie, Shi, & Zhang, 2017). However, no mediation effect of the brain's integration capabilities could be verified in this study, due to the small percentage of participants demonstrating signs of depression (N = 20).

### 4.3 PSMD versus WMH as surrogate markers for CSVD

Previous studies using diffusion weighted imaging (DWI) report that CSVD patients exhibit a higher mean diffusivity (MD), meaning an increased diffusion magnitude, as well as a decreased fractional anisotropy (FA), suggesting a decreased directionality of diffusion (van Norden, van Uden, de Laat, van Dijk, & de Leeuw, 2012; Tuladhar, Dijk, et al., 2016a). These findings might reflect a decreased fibre integrity and pathologic aggregation of free water in the extracellular compartment (Duering et al., 2018) and indicate that DWI is able to reflect microstructural changes caused by CSVD. Based on comparison of the linear models R^2^ values, our analysis suggests a superior explanatory power of PSMD compared to the different WMH loads with regard to the global efficiency, clustering coefficient and small‐world propensity. This observation may be owed to the robust calculation of the PSMD in contrast to the error‐prone nature of WMH segmentation. The algorithm we used for the automated segmentation of WMH tends to underestimate dWMH (Griffanti et al., 2016), potentially blurring the influence of WMH load on the topological network properties. As mentioned before, the PSMD might be more sensitive to rather subtle microstructural pathologies. The moderate CSVD burden in our study sample might therefore favour the PSMD, as the median WMH volumes were rather low compared to other cohort studies investigating CSVD. These results accentuate the PSMD's high capability of capturing CSVD severity ‐ even in brains appearing healthy on conventional MRI (Baykara et al., 2016).

### 4.4 Methodological considerations on structural connectome reconstruction

Weighted structural connectomes were successfully reconstructed in all participants.

Our connectomes exhibited a rather high density with regard to comparable previous studies (Lawrence et al., 2014; Tuladhar, Dijk, et al., 2016a; Tuladhar, van Uden, et al., 2016b) which is owed to our usage of the “2nd order integration over fibre orientation distributions 2”‐algorithm (iFOD2) (Tournier, Calamante, & Connelly, 2010) during tractography without a separate thresholding step (Civier, Smith, Yeh, Connelly, & Calamante, 2019). Tract‐tracing studies of macaque brains yielded similarly densely connected connectomes suggesting biological plausibility and accuracy of the type of connectome used in this study (Markov et al., 2011).

### 4.5 Strength and limitations of our study

Briefly, advantageous features of this work lie in the utilisation of PSMD as a CSVD surrogate parameter, the size of the sample and a state of the art and reproducible processing pipeline applied to it.

Our study has limitations. Even though the cohort represents a population with increased cardiovascular risk, the overall analysed participants were relatively healthy regarding imaging findings of CSVD. Findings might be different in a more severely affected sample. However, we would argue that our findings in a group of patients with relatively mild degree of CSVD point towards changes in white matter microstructure and disturbances of brain network topology already detectable at an early stage of CSVD. As we think of CSVD as a chronic‐progressive disease, it is likely that other graph parameters calculated in this study would follow characteristic trajectories depending on disease progression. Extrapolation to datasets with patients in more advanced stages of CSVD would most certainly be possible for the Clustering Coefficient, which is strongly dependent on overall connectivity strength (reflecting progressing loss of structural white matter integrity in CSVD). However, Modularity and Small World Propensity are parameters in graph theory more sensitive to topological network changes (specific anatomical pattern of white matter degeneration). Therefore, hypotheses regarding the translation of findings in our group to more severely affected patients cannot be tested validly in the current sample.

## 5 CONCLUSION

To summarise, in a large sample of subjects with vascular risk factors we were able to demonstrate that CSVD is associated with alteration of structural brain network topology considered crucial for maintenance of proper brain function – independently from the used surrogate parameter. Higher burden of CSVD goes along with a shift of brain network topology towards reduced integration, which might reflect the pathology underlying impaired cognitive function in CSVD and an increased segregation and consequently altered small‐world structure. These findings might reflect a pathology of the brain network functionality in CSVD that could explain the associated sequelae. Since the clinical impacts of increased segregation are not well understood yet, these parameters might serve as a promising subject for future studies in CSVD patients.

## CONFLICT OF INTEREST

Dr. Thomalla reports receiving consulting fees from Acandis, grant support and lecture fees from Bayer, lecture fees from Boehringer Ingelheim, Bristol‐Myers Squibb/Pfizer, and Daiichi Sankyo, and consulting fees and lecture fees from Stryker; Dr. Gerloff, receiving lecture fees and advisory board fees from Boehringer Ingelheim. Dr. Fiehler, receiving consulting fees from Acandis, Cerenovus, Medtronic, Microvention, Penumbra, and Route 92 Medical.

## AUTHOR CONTRIBUTIONS

We describe contributions to the paper using the CRediT contributor role taxonomy. Conceptualization: BF, MP; Data Curation: BF, MP, CM; Formal analysis: BF, MP, ES; Investigation: all; Methodology: BF, MP, ES; Software: BF, MP, ES; Supervision: GT, BC; Visualisation: BF, MP; Writing – original draft: BF, MP; Writing – review & editing: BF, MP, ES, CM, GT, BC.

## Data Availability

Due to the nature of this research, participants of this study did not agree for their data to be shared publicly, so supporting data is not available.
